# Actinomycin-X2-Immobilized Silk Fibroin Film with Enhanced Antimicrobial and Wound Healing Activities

**DOI:** 10.3390/ijms24076269

**Published:** 2023-03-27

**Authors:** Wenjing Zhou, Zhenxia Xie, Ranran Si, Zijun Chen, Ansar Javeed, Jiaxing Li, Yang Wu, Bingnan Han

**Affiliations:** 1 Laboratory of Antiallergy Functional Molecules, Zhejiang Key Laboratory of Silkworm Bioreactor and Biomedicine, College of Life Sciences and Medicine, Zhejiang Sci-Tech University, Hangzhou 310018, China; 2 School of Materials Science and Engineering, Zhejiang Sci-Tech University, Hangzhou 310018, China; 3 College of Textile Science and Engineering (International Institute of Silk), Zhejiang Sci-Tech University, Hangzhou 310018, China

**Keywords:** actinomycin X2, immobilization, antimicrobial silk fibroin film, biocompatibility, wound healing

## Abstract

Actinomycin is a family of chromogenic lactone peptides that differ in their peptide portions of the molecule. An antimicrobial peptide, actinomycin X2 (Ac.X2), was produced through the fermentation of a *Streptomyces cyaneofuscatus* strain. Immobilization of Ac.X2 onto a prepared silk fibroin (SF) film was done through a carbodiimide reaction. The physical properties of immobilized Ac.X2 (antimicrobial films, AMFs) were analyzed by ATR-FTIR, SEM, AFM, and WCA. The findings from an in vitro study showed that AMFs had a more broad-spectrum antibacterial activity against both *S. aureus* and *E. coli* compared with free Ac.X2, which showed no apparent strong effect against *E. coli.* These AMFs showed a suitable degradation rate, good hemocompatibility, and reduced cytotoxicity in the biocompatibility assay. The results of in vivo bacterially infected wound healing experiments indicated that wound inflammation was prevented by AMFs, which promoted wound repair and improved the wound microenvironment. This study revealed that Ac.X2 transformation is a potential candidate for skin wound healing.

## 1. Introduction

Antimicrobial peptides (AMPs) are currently receiving attention as an alternative to conventional antibiotics [1]. Compared with other compounds, peptides exhibit unique properties; their importance was discovered in 1922 when insulin was isolated from the pancreas of animals. As different synthetic strategies continue to be developed, such as studies on oxytocin in 1953 [2] and vasopressin in 1958 [3] which were performed by solution phase peptide synthesis strategies, synthetic human insulin was obtained by recombinant technology in 1982, leuprolide in 1985, and goserin in 1989. Various synthetic strategies for complex peptides were developed as an effective, efficient, and harmless alternative to small molecule drugs [4]. On the other side, the local application and short residence time of free AMPs are affected by environmental sensitivities such as hydrolysis, oxidation, enzymatic hydrolysis, photolysis, and pH. Therefore, AMP formulations are essential to improve stability, extend delivery time, and optimize the effectiveness at the application site [5]. Peptide chain cyclization is a way to improve the hydrolytic stability of AMPs [6]. Cyclic AMPs are mostly the result of artificial modification, such as L to D isomerization and intramolecular disulfide bond formation between cysteine residues, which have been adopted to enhance AMP stability against a range of host and microbe proteases [7]; in the meantime, the support of certain peptide structure transformation technologies is needed. Actinomycins, a family of chromogenic cyclic peptide antibiotics that differ only in the peptide portion of the molecule, were first discovered by Waksman and Woodruff in 1940 [8,9]. To date, among more than 30 discovered natural and synthetic analogs, actinomycin D has been the most widely researched and clinically applied due to its excellent antibacterial, antitumor, and antiviral activities [10]. Actinomycin D has been shown to maintain structural stability in different organic solutions, temperatures, and salt solutions. In our previous study, actinomycin X2 (Ac.X2) was obtained from the fermentation of a strain of *Streptomyces cyaneofuscatus*, and has similar stability [11]. In a previous research [12], Neutral Red (NR) has a similar amino group to our Ac.X2 chromophore, containing primary amino groups on the benzene ring. These amino groups increase EDC/NHS linkages, promoting the binding of NR to materials and enhancing the chemical as well as physical properties of composite material for stable immobilization of NR. In addition, Ac.X2 has shown effective antibacterial activity against Gram-positive bacteria while being less effective against Gram-negative bacteria [13] and its antibacterial activity was significantly better than that of actinomycin D [14,15]. On the other hand, it also exhibited potent anti-tuberculosis activity; however, its application is limited due to the potential cytotoxicity [16,17].

To reduce the cytotoxicity and enhance the antimicrobial stability of AMPs, they are often immobilized onto the surface of specific materials [18,19]. Considering its excellent biocompatibility, versatility and low immune response, silk fibroin (SF) is often regarded as one of the best candidates for tissue engineering. In the application of composite materials containing SF, numerous studies on promoting wound repair and inhibiting biofilm formation have been reported. SF is rich in active groups (e.g., carboxyl, hydroxyl, amine, etc.) that can be functionalized [20]. Among the many methods, the carbodiimide method is of interest because it is simple and involves an effective coupling agent such as EDC/NHS and Ac.X2 has a primary amino group, which fits well with the structure of SF [21].

In this study, Ac.X2 was immobilized to the surface of SF film through a carbodiimide reaction. The physical properties of these AMFs were analyzed by ATR-FTIR, SEM, AFM, water contact angle (WCA), etc. The antibacterial effects and biocompatibility were also evaluated. Finally, the potential application value was explored in an in vivo wound repair model, aiming to break through the clinical limitations of actinomycins and expanded their potential applications.

## 2. Results and Discussion

### 2.1. Preparation of Actinomycin-X2-Immobilized Silk Fibroin Film

**Figure 1:**
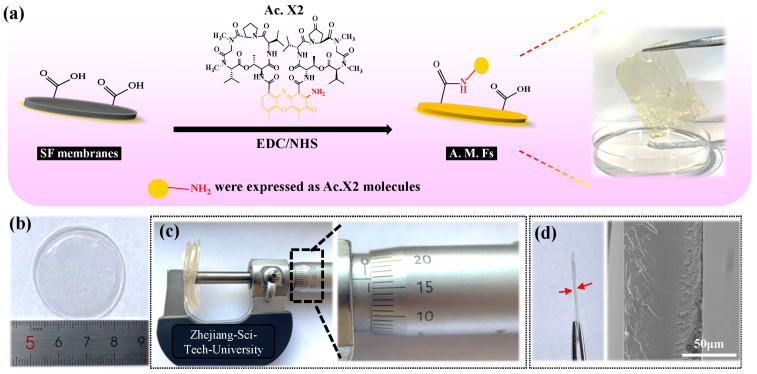
Preparation schematic diagram and three-dimensional data analysis of AMFs. (**a**) Schematic model for conjugation of Ac.X2 onto SF films by employing EDC/NHS; (**b**) showed that diameter of AMFs was 3.5 cm; (**c**) 10-fold thickness of AMFs is 666 μm using micrometer; (**d**) SEM images showed the thickness of a single film. The red arrow indicates the observed thickness of the SF film.

We soaked SF films in different initial concentrations (1 μg/mL, 5 μg/mL, 10 μg/mL, 20 μg/mL) of an Ac.X2 solution and used a carbodiimide reaction (EDC/NHS) to obtain Ac.X2-immobilized antibacterial films (AMFs). Table S1 shows the IDs of the different AMFs (A-1, A-5, A-10, A-20). The carbodiimide reaction can activate the carboxyl group of SF to couple with the primary amino group of the polypeptide to form a conjugate. The mechanism diagram in Figure 1a shows the binding principle of the carboxyl group of SF and the primary amine group of Ac.X2. To accurately quantify the loading of Ac.X2, the loading of Ac.X2 was converted into density of immobilized peptide; the measurement of three-dimensional data were completed by a micrometer caliper (accuracy of 10 μm), and the cross section thickness calculated by SEM was between 60 and 90 μm (Figure 1b–d). The thickness of the AMFs was similar to the skin substitute Suprathel (70~150 μm) that has a good impact on wound healing [22].

### 2.2. Characterization of AMFs

**Figure 2:**
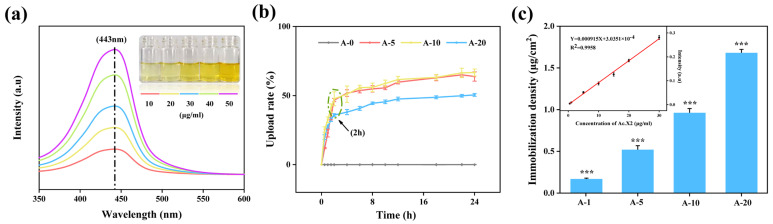
(**a**) UV–Visible spectra of different concentrations of Ac.X2; the strongest absorption peak was observed at 443 nm. (**b**) Uploading rate of SF films immersed in Ac.X2 solutions over time up to 24 h. (**c**) Immobilized peptide density of AMFs treated with different initial concentrations of Ac.X2 solution; immobilization density range of 0.16~1.67 μg/cm^2^. Standard linear curve among different concentration of Ac.X2 at 443 nm in range of 0.5~30 μg/mL (*** *p* < 0.001).

Evaluation of Immobilization. As shown in Figure 2a, Ac.X2 concentration and UV–Visible absorption intensity of Ac.X2 at 443 nm had a positive correlation in the reaction system. Ac.X2 has a phenoxazinone chromophore which is more accurate for quantification of free peptides instead of using colored chemical reactions. Considering that Ac.X2 has neither the long-chain terminal amino group nor a weak steric effect, the time of its immobilization was increased to 24 h to determine the optimal reaction time point. The curve of the uploading rate maintained a continuous upward tendency during reaction period of 24 h. The curve was in a rapidly rising stage within 2 h, but the upward tendency gradually leveled off after 4 h (Figure 2b). We believe that the chemical grafting reaction of Ac.X2 takes a dominant role in rapidly rising stage within the first 2 h, while the physical adsorption of Ac.X2 may be the main reason for the flat stage. Bai et al. [23] also proved that an approximate time of 2 h was the optimal reaction time for AMP immobilization on SF films. Therefore, we chose a time of 2 h as the reaction point. The uploading rate of A-5, A-10, and A-20 were 46.73 ± 1.17%, 47.68 ± 5.09%, and 35.91 ± 1.01%, respectively. The immobilization density of the different AMFs was determined from A-1 to A-20 in the range of 0.16~1.67 μg/cm^2^ (Figure 2c); the immobilized density can effectively reduce cytotoxicity while ensuring good antibacterial activity, which was demonstrated in our subsequent assays.

The comparison of physically absorbed Ac.X2 and covalently attached Ac.X2 (covalently attached Ac.X2/immobilized Ac.X2/AMFs) is shown in Figure S1.

**Figure 3:**
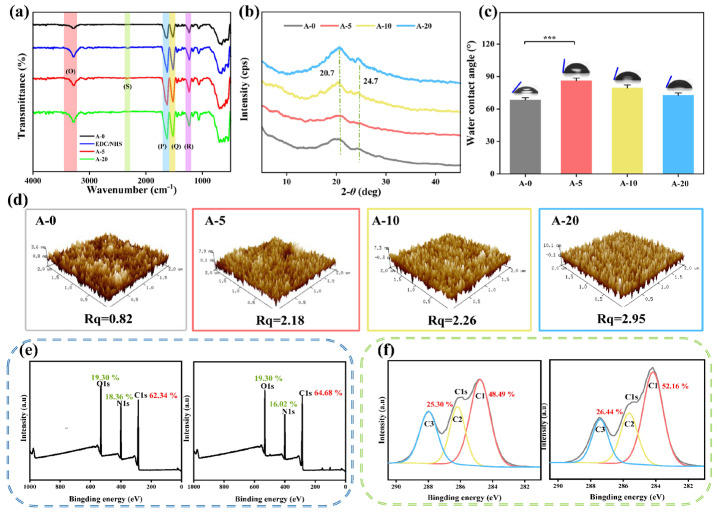
Surface characterizations analyzed by ATR-FTIR, XRD, water contact angle (WCA), AFM, and XPS. (**a**) O, P, Q, R, and S represented wavenumbers of 3225 cm^−1^, 1625 cm^−1^, 1520 cm^−1^, 1239 cm^−1^, and 2500~2250 cm^−1^, respectively. (**b**) XRD patterns and (**c**) WCA of different AMFs (*n* = 3, mean ± SD). Surface topographical morphology and roughness of A-0, A-5, A-10, and A-20 are shown in (**d**); XPS analysis results for characterization of surface elemental composition, survey scan spectra are shown in (**e**), and high resolution C1s spectra are shown in (**f**) (*** *p* < 0.001,). Note: The blue column is control (A-0) and green column is A-20.

Evaluation of ATR-FTIR. The analysis of protein secondary structures by Fourier transform infrared spectroscopy (FTIR) is an effective and universal method [24]. In general, the spectral region between 1700 cm^−1^ and 1500 cm^−1^ is the absorption region of amide I and amide II groups, while the amide III region is between 1350 cm^−1^ and 1200 cm^−1^ [25]. SF films had absorption peaks due to C=O stretching vibrations (P, 1625 cm^−1^) of amide I, and N-H stretching (O, 3225 cm^−1^) and bending vibrations (Q, 1520 cm^−1^) of amide II, which were characteristic for silk I conformations (Figure 3a), and amide III was associated with C-N stretching vibrations (R, 1234 cm^−1^). Compared with A-0, A-5, and A-20, the transmittance of O, P, Q, and R showed an obvious downward trend, indicating that the Ac.X2 was successfully immobilized on the films (Figure S2). At the same time, we observed that EDC/NHS-modified films had a weak and broad peak at S (2500~2250 cm^−1^), which was the result of the combination with an ammonium salt [26]. With increase in concentration of Ac.X2, the peak disappeared indicating that the amine salt was replaced by Ac.X2. Therefore, we believe that Ac.X2 was successfully immobilized onto the SF film.

Evaluation of XRD. XRD is often used to study the crystallinity of silk fibroin [27]. The ordered α-helices and coils belong to the major structural conformations of silk I with diffraction angles of 24.7°, and 28.2° and 27.9°, respectively. Silk II structures are mainly β-sheet structures, with peaks corresponding to around 20.4°, 24.1°, 25.6°, and 30.9° [28,29]. When the Ac.X2 was added to the SF film surfaces, the characteristic peak of 24.7° belonging to silk Ⅰ gradually became prominent among A-0, A-5, A-10, and A-20, indicating that the Ac.X2 exhibited a steady state with silk fibroin in the form of silk Ⅰ (Figure 3b).

Evaluation of water contact angle (WCA) and AFM. Hydrophilicity and surface roughness are some of the factors to evaluate wound dressings. Previous studies have shown that material surfaces with high roughness may increase cell adhesion [30], and with suitable hydrophilicity are conducive to cell growth [31,32]. The WCA of A-0 was 68.27 ± 2.22°, and when the Ac.X2 was added, the surface hydrophilicity changed significantly; A-5, A-10, and A-20 had WCAs of 86.07 ± 2.51°, 79.33 ± 2.78°, and 72.57 ± 2.30°, respectively (Figure 3c). The result of A-0 to A-5 was mainly due to the blocking of a hydrophilic group of SF (such as carboxyl) and aggregation of the hydrophobic cyclic pentapeptides of Ac.X2 molecules [33]. Figure 3d shows that the surface roughness increased with the increase in Ac.X2 (A-0, Rq = 0.82; A-5, Rq = 2.18; A-10, Rq = 2.26; A-20, Rq = 2.95). In terms of this trend in A-5, A-10, and A-20, combined WCA and roughness model diagrams were consistent with Wenzel’s theoretical model. On the hydrophilic surface, with increased roughness, the hydrophilicity was also increased, leading to a smaller WCA [34]. Meanwhile, this tendency was in accordance with Zhao et al.’s [35] description of surface hydrophobicity and roughness. Zhang et al. [36] found that the behaviors of cell attachment and proliferation mainly rely on the surface properties of the material. Our experimental results also demonstrate the feasibility of using the tunable microstructure of Ac.X2-treated SF films as biomedical materials.

Evaluation of XPS. As shown in Figure 3e, changes observed between C1s clearly increased (62.34% → 64.68%) and N1s slightly decreased (18.36% → 16.02%) from A-0 to A-20, while no significant changes were observed in O1s (19.30%). Meanwhile, we analyzed the high resolution of C1s by peak fitting (Figure 3f), and both peaks of C1 (48.49% → 52.16%) at 285.09 eV for C-C or C-H carbon and C2 (25.30% → 26.44%) at 286.48 eV for C-N or C-O carbon increased in the composition of atoms [37]. As the Ac.X2 molecule contains remarkable carbon elements, the signal strength of N1s was relatively reduced when C1s signal strength increases, and the same result was observed for the N/C ratio (0.29 → 0.25) [38], thus indicating that Ac.X2 was immobilized on the surface of the silk fibroin. In addition, the dissolve–loss rate and swelling rate of AMFs were approximately 2% and 50%, respectively (Figure S3).

Thermal analysis of AMFs. Thermal analysis is one of the most important techniques to characterize the macroscopic physical properties of materials due to its ability to reveal the microphase structures for various materials [39]. Three thermal events (Figure S4a,b) appeared in all samples: the dehydration from ambient temperature to 100 °C, glass transition at a range of 150 °C to 250 °C, and decomposition was seen after 250 °C [40,41]. The DSC results (Figure S4c) showed that the endothermic peak decomposition of all samples treated by Ac.X2 changed slightly to lower temperatures. These results indicate that Ac.X2 can affect the mobility of silk fibroin chains.

Mechanical properties of AMFs. To mimic the typical water swelling conditions in a biological environment, all samples were first immersed in deionized water at 37 °C for 30 min before the mechanical properties test. The stress of silk fibroin films treated with Ac.X2 was significantly improved (Figure S4d) when compared with the untreated film (A-0). The stress of SF films treated with different Ac.X2 concentrations was about 0.97 MPa (A-0), 5.40 MPa (A-5), 5.97 (A-10), and 7.68 MPa (A-20). The elongation ratio of AMFs was 9.23% (A-0), 16.51% (A-5), 35.69% (A-10), and 67.26% (A-20). In general, the strength and elongation of each material were contradictory. If the material was rigid (stress), then the ductility (strain) of the sample was poor [42]. It is well known that the elongation rate at break is determined by the arrangement of the molecular chains of the protein [40]. Therefore, there may be two reasons for this phenomenon: (1) Ac.X2 helps a part of the amorphous region of the SF chain to form a more ordered orientation, which is also shown in the XRD results; and (2) in the process of Ac.X2 solution treatment, water molecules had a strong plasticization effect on the molecular chains of silk cellulose.

### 2.3. Evaluation of Antibacterial Activity

**Figure 4:**
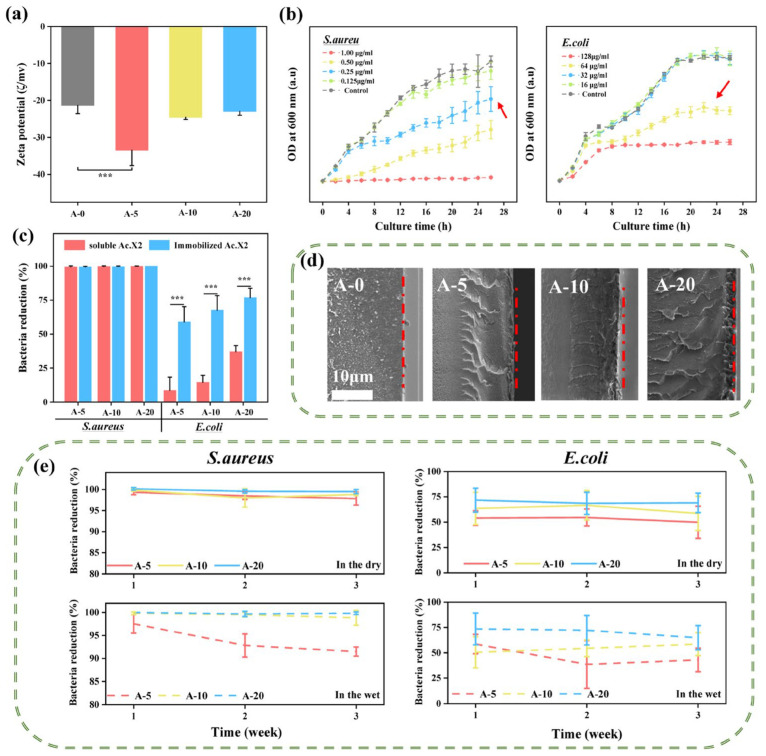
Evaluation of in vitro antimicrobial activity of AMFs. (**a**) Zeta potential of A-0, A-5, A-10, and A-20; (**b**) minimum inhibitory concentrations of Ac.X2 against *S. aureus* and *E. coli* by broth microdilution method. MIC for *S. aureus* was 0.25 μg/mL and 64 μg/mL for *E. coli*. The red arrow indicates the corresponding curve of the MIC. (**c**) Antibacterial activity with immobilized Ac.X2 compared with same amount of soluble Ac.X2; (**d**) SEM images of cross sections of 2 h-immobilized Ac.X2 SF films from A-0 to A-20. The red line indicates the interface between the sample surface and air. (**e**) Long-term antibacterial activity of AMFs against *S. aureus* and *E. coli* (—: in a dry environment, ---: in a wet environment). Bacterial reduction (%) was calculated based on colony forming unit (CFU) of pristine SF (*n* = 3, mean ± SD). Note: *** *p* < 0.001.

The initial adhesion and subsequent bacterial growth are often closely related to surface roughness, energy, and potential. Antibacterial agents containing anions can reduce the biological adhesion of biomaterials [43]. Hydrophilic surfaces can repel bacterial adhesion by reducing contact angles and increasing wettability. However, when the contact angle was similar, the surface material with a negative charge was more resistant to adhesion, because most bacteria have negatively charged cell walls [44,45]. AMFs showed overall electronegativity with A-0, A-5, A-10 and A-20 having zeta potentials of −21.32 ± 1.21 ζ/mv, −33.48 ± 4.16 ζ/mv, −24.58 ± 0.57 ζ/mv, and −22.97 ± 1.04 ζ/mv, respectively (Figure 4a); this was one of important influencing factors in preventing bacteria from forming biofilms on SF films.

The MIC (the minimum concentration to inhibit bacterial growth) of *E. coli* and *S. aureus* by the broth microdilution method [46] were 64 μg/mL and 0.25 μg/mL (Figure 4b and Figure S5a). These two strains have been frequently associated with human infections. The antimicrobial activity of immobilized Ac.X2 and soluble Ac.X2 against *E. coli* and *S. aureus* are presented in Figure 4c; as immobilization density increased, anti-bacterial activity (%) also increased.

In terms of the *S. aureus* strain, Ac.X2 solutions with initial concentrations of 0.25 μg/mL (MIC), 0.5 μg/mL, 1 μg/mL, 5 μg/mL, 10 μg/mL, and 20 μg/mL were selected to identify the immobilization density with lowest effective antibacterial effect against *S. aureus.* Against *S. aureus,* the activity of A-0.25, A-0.5, and A-1 are shown in Figure S5b. Neither soluble Ac.X2 nor immobilized Ac.X2 had excellent antibacterial activity. This may be due to the rupture of film surface during the reaction (Figure 4d), which led to Ac.X2 molecules entering into the film, forming a direct contact with bacteria at a low concentration of Ac.X2, which resulted in more unstable antibacterial properties. Soluble (free) Ac.X2 amounts equal to A-5, A-10, and A-20 and immobilized Ac.X2 resulted in over 99% bacterial reduction of *S. aureus*. Agar plates pictures of *S. aureus* treated with different AMFs is shown in Figure S5c.

In terms of an *E. coli* strain under the same amount of Ac.X2, the immobilized Ac.X2 produced stronger antibacterial activity than soluble Ac.X2; the bacterial reduction of A-5, A-10, and A-20 were 58.93 ± 11.26%, 67.67 ± 10.60%, and 76.90 ± 6.85%, respectively, while the soluble Ac.X2 was 8.76 ± 3.62%, 14.62 ± 5.09%, and 37.04 ± 4.58%, respectively. Because *E. coli* have physical appendages (e.g., flagellum exhibits a variety of adhesins [47]) that allow it to move spontaneously, the SF film acted as a substrate for bacterial anchoring [48]. Bacteria gathered on film surfaces through physical adsorption [49] (reversible adsorption) or chemical adsorption [50] (passive or active), came into contact with local high concentrations of Ac.X2, which was different from the uniform distribution of soluble peptides, and produced an inhibitory effect. Therefore, immobilized AMFs showed the capacity to adsorb bacteria and kill them.

In general, the antimicrobial activities of immobilized AMPs were significantly lower than those of soluble AMPs [43]. This change in bacteriostatic properties due to AMPs embedded in the material was similar to the results of many previous studies [51,52]. However, immobilized Ac.X2 exhibited intense antimicrobial activity against *E. coli* which was sharp contrast to soluble Ac.X2. Furthermore, the overall trend of AMP content and increase in maximum antibacterial rate showed that the immobilized Ac.X2 induced antibacterial effects on the material surface through leveling of immobilization density. This phenomenon can be explained by correct peptide orientations, increasing flexibility of linkers, and density of immobilized peptides [43]. First of all, for straight chain antimicrobial peptides, consistency and effectiveness of antibacterial activity generally require a peptide sequence equal to or greater than 10 amino acids [51]. The length of Ac.X2 was smaller than that of its own straight chain form due to the closed structure of the head and tail ends. Afterwards, Ac.X2 molecules were planar molecules, and their parallel or vertical arrangements had a certain effect on the activity of its accessible surface, and this may be related to their antibacterial ability. Furthermore, EDC/NHS as a linker was not introduced to any space (between two-carbon or a six-carbon chain linker) [1,51]. Therefore, the process of increase or fluctuation of bacteriostasis caused by linker addition was excluded. Finally, it was speculated that this phenomenon was due to the bacteriostatic effect of immobilized Ac.X2 at a high density against *E. coli* different from a uniformly dispersed solution of soluble peptides.

The long-term antimicrobial properties of Ac.X2 against *S. aureus* and *E. coli* are shown in Figure 4e, which showed that after three weeks, the antibacterial effect still retained. This result is attributed to the anchoring of Ac.X2 to the SF film, with the formation of secondary bonds between the SF film and Ac.X2 strengthening the long-term antibacterial activity of the AMFs.

### 2.4. Bactericidal Mechanisms of AMFs

**Figure 5:**
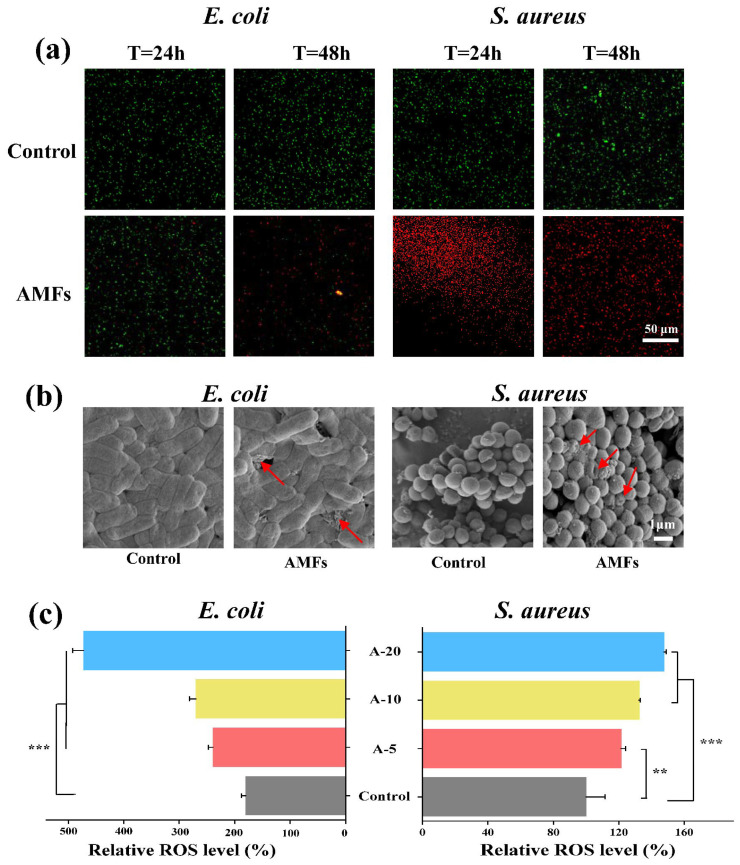
Bactericidal mechanisms of AMFs. (**a**) Live/dead staining results of *S. aureus* and *E. coli* incubated with AMFs. Biofilm incubated with A-0 was used as control. (scale: 50 µm); (**b**) SEM image of *S. aureus* and *E. coli* treated with control and AMFs (scale: 1 µm); (**c**) ROS released by interaction of antibacterial agents with culture medium. Red arrows indicate damaged bacteria. (*** *p* < 0.001, ** *p* < 0.01).

SYTO9 can penetrate the cell membrane of bacteria and stain the cells green, while PI can only penetrate dead cells with damaged cell membranes and stain the cells red [53]. The results of the live/dead staining are shown in Figure 5a. After 24 h and 48 h, *S. aureus* treated with AMFs always showed a red fluorescence, even though the green fluorescence intensity of control group increased. *E. coli* treated with AMFs also showed a predominance of red fluorescence with increasing time, which suggest a reduction in cell viability. Bacterial biofilms can form within hours of contact between planktonic bacteria and medical implants [49]. As the first few hours are crucial for biofilm formation, the AMFs were used to analyze the changes in cell membrane morphology at 3 h (*S. aureus*) and 12 h (*E. coli*) in vitro. Live/dead staining was used in combination with morphological visualization (Figure 5b), which showed the possibility of cell apoptosis through the phenomena of lysis and shrinkage of the cell walls.

The ROS are released by bacteria after interaction with antibacterial agents, and ROS are one of the main factors for antibacterial action [54,55]. Lin et al., found that ROS and H_2_O_2_ have a dose-dependent effect on the content of actinomycin D [54]. It has been reported that changes in ROS in bacteria are mainly caused by the autoxidation of NADH dehydrogenase II in the respiratory chain [56]. It irreversibly damages bacteria (e.g., cell membrane, DNA, mitochondria, etc.), resulting in bacterial cell death [55]. Figure 5c shows the ROS release level after interaction of AMFs and bacteria. The relative ROS release level of *S. aureus* and *E. coli* ranged from 100.01 ± 11.63 to 147.71 ± 1.38% and 180.05 ± 7.99 to 472.21 ± 19.83%, respectively, and was dependent on Ac.X2 concentration.

Most of these linear antimicrobial peptides were derived from organisms and belong to cationic antimicrobial peptides [57]. Their antibacterial mechanism can be explained by classical electrostatic adsorption. According to molecular radius calculations [58] and a description proposed by Hu [59], it was observed that the molecular three-dimensional structure of Ac.X2 had a length less than 20 nm and dimensions of 2.45 × 1.65 × 1.23 nm, and the thickness of bacterial cell wall is 20~80 nm [44]. Immobilized Ac.X2 neither penetrates the cell wall nor enters inside the cell. Compared with positive antimicrobial peptides, AMFs had no characteristics negative electrostatic adsorption with bacterial surfaced to produce film instability and kill the bacteria [60]. Finally, combined with the above factors, we speculated that the antibacterial mechanism of Ac.X2 was due to the increase of peptide density on the surface, which induced apoptosis within bacteria [54]; however, its specific antibacterial mechanism remains to be further verified [61].

### 2.5. Evaluation of Biocompatibility

**Figure 6:**
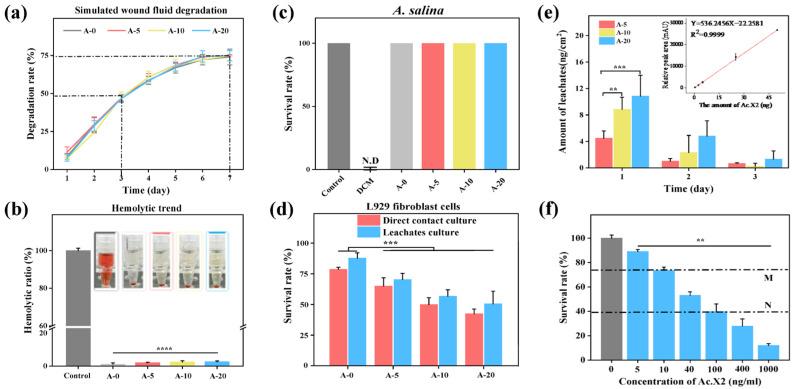
Biocompatibility evaluation of AMFs. (**a**) Degradation behavior of different AMFs in simulated wound fluid containing Protease XIV; (**b**) hemolysis rate of AMFs and representative images of hemolysis results; (**c**) survival rate of *A. salina* by direct contact culture method (N.D = Not detect); (**d**) survival rate of L929 fibroblast cells cultured with direct contact culture method (red column, 65.02~42.41%) and leachate culture method (blue column, 70.30~50.63%) for 24 h; (**e**) amount of different AMFs leachates obtained by HPLC analysis in 1, 2, and 3 days. Inserted graph represents the standard linear curve of the amount of Ac.X2 and relative peak area by HPLC; (**f**) survival rate of L929 fibroblast cells with different concentrations of Ac.X2. The MN interval represents survival rate of L929 cells corresponding to free Ac.X2 at concentrations ranging from 10 to 100 ng/mL. Grey columns indicate control containing culture medium only. Note: **** *p <* 0.0001, *** *p <* 0.001, ** *p <* 0.01.

First, we evaluated the biocompatibility of AMFs through various assays. Studies have shown that the enzymatic biodegradation process and biostability of biopolymer materials are relatively complex, which may be related to the polymer structure, surface morphology, and molecular interactions between polymer chains [62,63]. As seen in Figure 6a, with the increase in degradation time, the degradation rate of SF films slowed down, which could be efficiently controlled by altering the content of the β-sheets [64], indicating that different AMFs had rational biodegradability (at 7 days, 74.04~75.12%). Figure 6b represents the result of the blood compatibility test which met hemolytic rate criteria for medical materials (ISO 10993-4, less than 5%) and it did not produce a severe hemolytic reaction. The brine shrimp toxicity test is used for the preliminary exploration of safety [11]. The result of direct contact methods showed that the survival rate of brine shrimp was 100%, and different AMFs did not show biological toxicity to brine shrimp, which reflected that neither film itself nor the concentration of exudate had reached a lethal degree for brine shrimp (Figure 6c). The cytotoxicity of AMFs is important to evaluate to be considered as a candidate material for direct contact with skin wounds [65]. The cytotoxicity of different AMFs was further evaluated by a viability assay using L929 fibroblast cells. The cell viability range of the direct contact culture method (treated with A-5, A-10, and A-20) was 65.02~42.41% for 24 h, and that of the leachate culture method was 70.30~50.63% (Note: *** *p* < 0.001, ** *p* < 0.01, * *p* < 0.05) for 24 h (Figure 6d). There was no significant difference between the two culturing methods. At the same time, the survival rate of A-5 was over 70%; according to “ISO10993-5 Biological Evaluation of Medical Devices” [66], a cell survival rate of more than 70% is considered safe, meaning that A-5 has no potential cytotoxicity. The morphologies of L929 fibroblast cells on the AMFs for 24 h were analyzed using inverted phase contrast microscopy (Figure S6). When cultured on A-0 to A-20, L929 cells changed from a normal spindle shape to a retracted circle; cell spacing significantly increased and cell density decreased among these morphologies. This result more intuitively shows that A-5 has good cell growth safety.

Figure 6e shows the exudation change of Ac.X2 in AMFs over 3 days; additionally, a deep analysis of exudation amount was performed on first day for accurate assessment of cytotoxicity within 24 h. The amounts of leachates (fourth column) which did not reach 1% of immobilization density (first column) is mentioned in Table 1. The results showed that nanogram amounts of leachates entered into the solution system. The amounts of leachates (fourth column) was converted to concentration of leachates (fifth column) in AMFs (0.6 cm disc) which ranged from 25.39 ng/mL to 61.36 ng/mL. According to the survival rate histogram of different concentrations of Ac.X2 (Figure 6f), the corresponding concentration range of Ac.X2 in the MN range was 10 ng/mL to 100 ng/mL, indicating that the cell survival rate ranged from 73.57% to 39.97%, which corresponded with survival rates shown by above two culture methods. The results showed that the viability of L929 cells in the exudation of AMFs in the direct contact culture method (65.02~42.41%) and in the leachates culture method (70.30~50.63%) were both verified.

The highly specific inhibitory effect of Ac.X2 on DNA-dependent RNA synthesis requires cellular uptake and, after entry, binding to double-helix DNA by inserting into the chromophore and through hydrogen bonding and hydrophobic interactions in the peptide’s lactone moiety [67,68]. Immobilized Ac.X2 could not freely diffusion for cell uptake; thus, the reason for cell death was mainly due to free Ac.X2 molecules entering into the cells, while the mass of Ac.X2 molecules embedded in SF film was not the main reason for cytotoxicity. In general, surfaces combined with antibiotics or other biocidal substances have the advantage of delivering drugs directly to target site, resulting in locally high drug doses without exceeding the systemic toxicity level of the drug, and preventing harmful side effects [43].

Finally, according to the concentration–survival curve shown in Figure S7, the concentration-dependent change gradually flattened when the concentration was greater than 0.2 μg/mL, and when the concentration was greater than 1 μg/mL, the survival rate was less than 11.09%. Table 1 shows that the concentration of free Ac.X2 (third column) corresponding to the immobilized Ac.X2 (second column) was greater than 1 µg/mL; it was not difficult to speculate that the survival rate caused by free Ac.X2 was less than 11.09% without immobilization. Therefore, we conclude that immobilized Ac.X2 has reduced toxicity compared to free Ac.X2 and has good biocompatibility.

### 2.6. Evaluation of the Stability of AMFs

**Figure 7:**
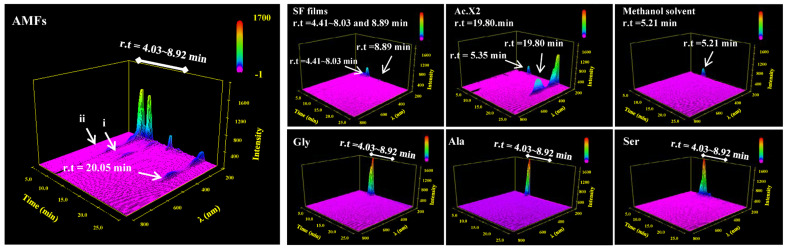
HPLC traces of pure substances were used to analyze the exudative Ac.X2 in AMFs. Likewise, there are absorption peaks (Gly, Ala and Ser) between r.t 4.03 and 8.92 min at 443 nm. HPLC traces of the three pure substances in SF films; Ac.X2 and methanol solution were also shown. i and ii may be debris peaks following covalent reactions between amino acids and Ac.X2. At a range of 190 nm < λ < 210 nm below the wavelength limit of methanol, the absorption peak of the solvent was exposed; the range of 250 nm < λ < 500 nm belonged to heteroatomic unsaturated groups (e.g., -C=O, -N=O, -N=N, etc.).

To evaluate the stability of immobilized Ac.X2, AMFs were incubated in physiological medium for 7 days at 37 °C and analyzed through HPLC using a diode UV detector within the range of 190~400 nm [69]. (Figure 7). The solvent peak of methanol was seen near the retention time (r.t) of 5.21 min. Ac.X2 had a maximum absorption peak near the r.t of 19.80 min. In addition, r.t 4.41~8.03 min and r.t 8.89 min were the fragment peaks after dissolution of the SF films. These were mostly small molecule amino acids shown by HPLC traces of the three most abundant amino acids (Gly, Ala, Ser) in the SF [20]. The absorption peak at 443 nm was regarded as the absorption peak of Ac.X2; we speculated that this fragment (i and ii) at r.t 4.03 to 8.92 min at 443 nm, was Ac.X2 peptide fragments after Ac.X2 was bound to SF film (in the AMF traces). Since peptides have polar groups, when they are separated using a C_18_ column and compared with pure Ac.X2, the retention time would be advanced (before 19.80 min). At the same time, the retention time of most amino acids was also within 4.03 to 8.92 min. Combined with our previous studies [11], it was believed that the structure of immobilized Ac.X2 was not damaged after gentle combining with SF films.

### 2.7. In Vivo Evaluation of Wound Healing

#### 2.7.1. Evaluation of Full-Thickness Skin Infected Wound Model

**Figure 8:**
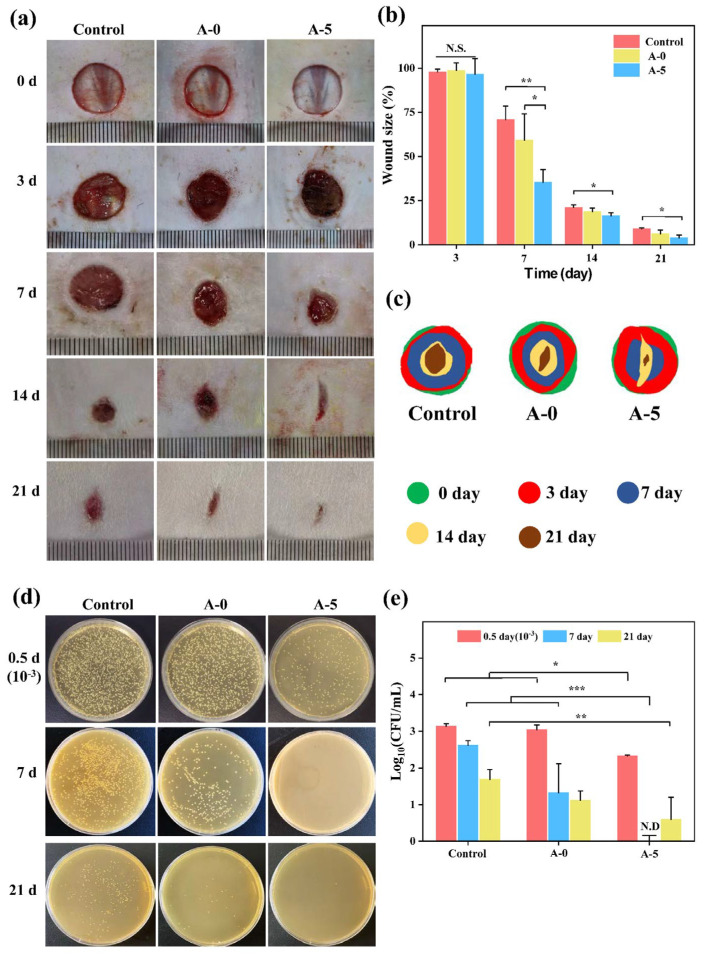
(**a**) Images showed wound healing at time points for each group (Control, A-0, and A-5); (**b**) Wound size at 3, 7, 14, 21 days; (**c**) Wound dressing effect was present based on full-thickness cutaneous defect mode; (**d**) In vivo antibacterial result of single colony map; (**e**) Antibacterial statistical analysis and 10^−3^ represented result after three times dilution. Note: *** *p <* 0.001, ** *p <* 0.01, * *p <* 0.05.

In the model of full-thickness skin wound healing, three groups including control (gauze), A-0, and A-5 were used to demonstrate the potential of AMFs [70,71]. As illustrated in Figure 8a, the defected skin was significantly different at days 3, 7, 14, and 21 among all groups. The wound size was quantified by ‘Image Pro Plus 6.0’ in Figure 8b. The group treated with A-5 showed a 35.43 ± 7.19% wound size in 7 days followed by 59.22 ± 14.95% and 70.90 ± 7.63% in A-0 treated and control (gauze), respectively. Furthermore, A-5 treatment show the fastest repairing effect in 21 days. These results indicate that A-5 has obvious advantages against *S. aureus* in the early stages of wound repair.

The agar map of colonies of the in vivo antimicrobial analysis are presented in Figure 8c,d shows that the bacterial infection in wounds treated by A-5 was significantly lower than that treated by A-0 and control after 0.5 d (10^−3^ represents bacterial solution diluted three times), 7 d, and 21 d. Statistical analysis of antibacterial activity is presented in Figure 8e. Log_10_ (CFU/mL) of gauze (control), A-0, and A-5 were 2.62 ± 0.11, 1.33 ± 0.79, and 0 after 7 days, respectively. This phenomenon indicated that A-5 has good in vivo antibacterial activity. Considering the above results, AMFs (A-5) have better antibacterial effects and wound healing effects in vivo.

#### 2.7.2. Histological and Immunohistochemical (IHC) Evaluation

**Figure 9:**
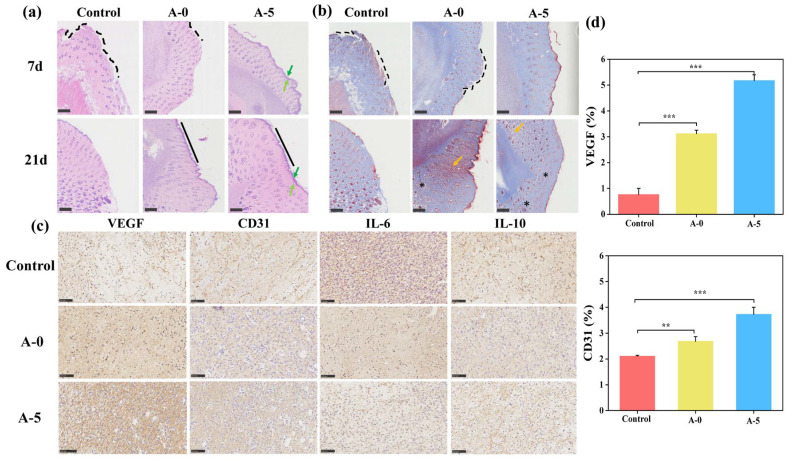
Histological and immunohistochemical wounds staining. H&E (**a**) and Masson (**b**) staining images of wounds treated with control (gauze), A-0, and A-5 on day 7 (scale: 500 μm).Dark green arrow shows cuticle layer of upper epidermis, light green arrow shows basal layer, black dashed line indicates a discontinuous epithelium, black straight line indicates continuous upper epidermis, orange arrow indicates muscle fibers, and * indicates collagen fibers. (**c**) VEGF, CD31, IL-6, and IL-10 immunohistochemical staining of wounds treated with gauze, A-0, and A-5 on day 7 (scale: 100 μm); (**d**) statistical analysis of differences in VEGF and CD31 levels (*** *p <* 0.001, ** *p <* 0.01).

H&E and Masson’s trichrome staining (Figure 9a,b) were used to compare epithelial tissue structure, collagen deposition, and maturation of the control, and A-0- and A-5-treated groups. The A-5 group had a relatively complete and continuous epithelial structure, and differentiation of cuticle and hair follicle formation had begun. A-0 had a discontinuity in epithelial formation and the control still had some unfilled gap problems and did not produce significant amounts of new epithelium. Such differences could be due to the bacterial infection preventing the repair of the upper epidermis, and the A-5 group containing Ac.X2 can overcome this problem early. At the same time, it was observed that there was large number of collagen fibers and muscle fibers, but the A-5 treated samples showed obvious high densities of collagen fibers [72,73], while the control did not, and the performance of muscle fibers was also lacking. On the other hand, SF has been reported to promote wound healing by activating the classic NF-κB signaling pathway [74], which has been associated with pro-inflammatory cytokines. Considering this, we performed further analysis by IHC.

Using immunohistochemistry (Figure 9c), the angiogenesis level was evaluated and the response of the wound microenvironment was validated. VEGF and CD31 are commonly used to assess angiogenesis, and IL-6 and IL-10 are commonly used to reflect inflammation [75]. The A-5 treated group showed the most intense staining of VEGF and CD31, indicating that the angiogenesis ability and platelet adhesion were optimal in a hematopoietic microenvironment [76]. The results of statistical analysis are presented in Figure 9d. By up-regulating the anti-inflammatory factor IL-10 and down-regulating the proinflammatory factor IL-6, it was found that control group had the most inflammatory microenvironment [77]. That is, AMFs greatly promoted the expressions of CD31 and VEGF, and \VEGF is a crucial growth factor in revascularization in wound healing [78]. It is generally recognized that VEGF promotes revascularization in skin wound repair, which is consistent with the staining for VEGF/CD31 in our study. The results showed that the A-5 treatment containing Ac.X2 had significant antibacterial potential, accelerated angiogenesis, improved the wound microenvironment, and promoted the healing of infected wounds.

## 3. Materials and Methods

### 3.1. Materials

*Bombyx mori* silkworm cocoons were purchased from Shiquan County, Shaanxi Province. Antimicrobial peptide Ac.X2 was produced by *Streptomyces cyaneofuscatus*, which was co-attached to cyanobacteria (*Lyngbya* species) collected from Nanji Island, Wenzhou, China. Cell culture reagents were purchased from Thermo Fisher Scientific, Waltham, MA, USA. All other unlisted chemical reagents were purchased from Macklin, Shanghai, China.

### 3.2. Preparation of AMFs

#### 3.2.1. Preparation of SF Films

Silkworm cocoons were treated with boiled 0.2% (*w*/*v*) sodium carbonate solution to remove the sericin. Degummed cocoons were washed thrice in deionized water and dried overnight at 65 °C. SF was obtained by dissolving it in a 9.3 M lithium bromide solution at 65 °C for 2 h. SF was dialyzed against purified water using dialysis bags (MWCO: 12~14 kDa) for 3 days. The SF solution was processed using a refrigerated centrifuge (TGL20M, JTLIANGYOU Co., Ltd., Jiangsu, China) to obtain a concentration of 3.0% (*w*/*v*) quantified by the mass analysis method:

Final concentration (%) = (Initial weight − final weight)/(Volume of solution) × 100(1)

The SF solution was casted onto polystyrene disks and dried at 25 °C with a relative humidity of 50% for 24 h. To obtain water-insoluble SF films, it was placed at 65 °C and 90% RH in a constant temperature and humidity incubator (HSP-80BE Ⅱ, Shanghai, China) for 3 h.

#### 3.2.2. Preparation of Purified Ac.X2

An actinomycetes seed solution was cultured with ISP4 medium at 28 °C and 180 rpm for 7 days. After the repeated fermentation, the supernatant was obtained by vacuum filtration and extracted by liquid–liquid extraction (ethyl acetate/fermentation liquor = 1:1) to retain the organic phase extraction layer, whose salt ion was removed by solid-phase extraction, and then low purity Ac.X2 was obtained. To improve the purity of Ac.X2 above 95%, the extraction liquid was processed using high-performance liquid chromatography (LC3000, Beijing Chuangxintongheng Science &Technology Co., Ltd., Beijing, China) after filtering through a 0.22 µm [11] cellulose membrane.

#### 3.2.3. Preparation of the Ac.X2-Immobilized SF Film 

Silk fibroin (SF) films were soaked in an MES (pH = 5.5~6.0) buffer containing EDC (0.8 mg/mL)/NHS (1.2 mg/mL) for 30 min at room temperature. After that, the activated SF films were transferred into phosphate-buffered saline (PBS, pH = 7.2) and mixed with different initial concentrations of Ac.X2 (1 μg/mL, 5 μg/mL, 10 μg/mL, 20 μg/mL), which were dissolved in methanol. Then, SF films were rinsed three times with deionized water to remove the excess solvent. The physically absorbed films without EDC/NHS were also obtained simultaneously.

### 3.3. Characterizations of AMFs

#### 3.3.1. Determination of Immobilized Peptide Density

To accurately quantify the loading of Ac.X2 molecules on antimicrobial films, the absorbance of the solution, before and after the reaction, was recorded to calculate the Ac.X2 loading on the films. The immobilization density and uploading rate were calculated by the following formulas:

Immobilized peptide = (Initial Conc. − Final Conc.) × (Volume of solution)(2)

Immobilization density = (Immobilized peptide)/(Surface area)(3)

Upload rate (%) = (Initial Conc. − Final Conc.)/(Initial Conc.) × 100(4)

The wavelength of the Ac.X2 solution was determined by an ultraviolet spectrophotometer (UV 2600, Shimadzu, Kyoto, Japan).

#### 3.3.2. Analytical Methods of Characterization

The ATR-FTIR spectra of the AMFs were recorded with a Nicolet iS20 attenuated total reflection-Fourier transform infrared instrument in the range of 400~4000 cm^−1^ with a resolution of 4 cm^−1^. The surface elemental compositions of A-0 and A-20 were measured by employing an X-ray photoelectron spectrometer (XPS) (K-alpha, Thermo Scientific, Waltham, MA, USA) equipped with a monochromatic Al Kα X-ray source (hv = 1486.6 eV). The surface morphology of AMFs was measured by Bruker Dimension ICON, a square pyramid in shape with a spring constant of 50 N/m, and a nominal radius of curvature of 10 nm. The samples were tested in triplicate. XRD was used to evaluate the crystallinity on the surface of the film materials. The sessile drop method was used for the measurement of the WCA (water contact angle). The thermal properties of samples were tested by using differential scanning calorimetry (DSC) and thermogravimetric analyses (TGA). The mechanical properties were tested using a tensile stress tester (KSM-BX5450ST).

### 3.4. Antimicrobial Activity Assessment

#### 3.4.1. Bacteriostatic Method

The antimicrobial activity was performed according to a modified JIS Z 2801 method. Using bacterial strains of *Staphylococcus aureus* (ATCC 6538) as Gram-positive and *Escherichia coli* (ATCC 8739) as Gram-negative representatives.

For the preparation of the original bacterial fluid, the strains that were stored in an LB agar plate (for less than two months), were transferred from the refrigerator (at 4 °C) into test tubes containing liquid broth. A single colony of each strain was isolated and subsequently cultured in 5 mL LB broth at 37 °C for 20 h. The original bacterial solution was diluted to 1 × 10^5^ CFU/mL, which was used for next step. To evaluate the antimicrobial activity of different AMFs against *S. aureus*, 50 μL diluted bacterial solution was inoculated on AMFs (20 × 20 mm^2^, sterilized by 70% alcohol and irradiated under ultraviolet radiation for 30 min), then flattened with a glass cover slip and incubated at 37 °C and 90% RH in a bacterial incubator for 24 h. After cultivation, the adherent bacteria on the film surface were repeatedly washed with 1 mL of PBS diluent until the surface was washed completely. In addition, to compare antimicrobial activity between soluble Ac.X2 and immobilized Ac.X2, we placed different AMFs and equal amounts of soluble Ac.X2 in 5 mL broth at 37 °C for 24 h. In both schemes, 1 mL of suspension was taken and 10-fold continuous dilutions were prepared and 1 mL of the diluted solution was inoculated onto LB agar plates. Then, the plates were incubated at 37 °C for 24 h and the bacterial reduction rate was calculated. A-0 was used as a control for comparison with different AMFs, and the calculation equation was as follows:

Bacteria reduction (%) = (A − B)/(A) × 100(5)

where A is number of colonies in the control and B is number of colonies on the samples.

#### 3.4.2. Long-Term Antimicrobial Stability Test

Wet store groups were soaked in PBS (37 °C) and dry store groups were sealed (25 °C) for 3 weeks. At each time point, an antimicrobial activity test was performed against *S. aureus* and *E. coli* with the previously described method. The above groups were kept in dark conditions.

### 3.5. Biocompatibility Assessment

#### 3.5.1. Brine Shrimp Culture

The biotoxicity of the antimicrobial films was determined using the number of brine shrimp survival rate; the positive control group (Dichloromethane, DCM) and negative control group (A-0) were set.

#### 3.5.2. Cell Culture

The evaluation of activity was performed by direct contact culture. The AMFs were trimmed to discs (D = 0.6 cm) and soaked in deionized water for 30 min to remove surface residues. A culture solution containing 1 × 10^4^ L929 fibroblast cells (iCell Bioscience Inc., Shanghai, China) was added to the 96-well culture plate after the discs were placed at the bottom and incubated in a humidified atmosphere at 37 °C containing 5% CO_2_ for 24 h; the blank control and negative control were set as the culture medium without cells and culture medium containing cells, respectively. The cell activity was evaluated using the CCK-8 assay, where six wells were set up for each sample at same time.

The assessment of leachate culture activity was consistent with the above method. Each 100 μL of DMEM medium contained the specified Ac.X2 amount in a 0.6 cm disc. It should be noted that cells need to be in an adherent culture for 12~18 h before co-culturing with leach liquor.

Survival rate (%) = (Experimental group − Blank control)/(Negative control − Blank control) × 100(6)

### 3.6. Assessment of Leaches

AMFs with clean and intact surfaces were immersed in a certain amount of physiological medium. AMFs were taken out repeatedly and rinsed with deionized water at each time point, the residual liquid was collected to quickly freeze in a −80 °C refrigerator and it was treated with vacuum freeze-drying for 24 h. The freeze-dried powder was dissolved in 400 μL methanol and transferred to a centrifuge tube through a 0.22 μm membrane filter. The content of antimicrobial peptide Ac.X2 was analyzed by HPLC (LC-16, SHIMADZU). Note: # Indicates that the film was a disc with a diameter of 0.6 cm. ## The concentration of exudative Ac.X2 and 100 μL culture medium after homogenization was calculated for comparison of cell viability.

Immobilized peptide # = (Immobilization density)/(Surface area)(7)

Free peptide concentration # = (Immobilized peptide #)/(Culture medium volume)(8)

Concentration of leachates ## = (Amounts of leachates)/(Surface area)/(Culture medium volume)(9)

### 3.7. In Vivo Wound Healing Assay

An SD rat wound model infected with *S. aureus* was used to study the wound healing properties of the AMFs [79]. SPF male SD rats (4~6 weeks, 200 ± 20 g weight) were provided by Beijing Huafukang Biotechnology Co., Ltd., Beijing, China. After administration of isoflurane anesthesia, a round full-thickness skin was defected with a diameter of 1 cm on the dorsal side. The wound was uniformly colonized with 10 μL *S. aureus* (10^8^ CFU/mL), and then it was closed for 2 days to establish an infected wound model. The rats were randomly divided into three groups (*n* = 6 per group): control group, A-0 group, and A-5 group. The wound sites were covered with gauze in the control group, whereas those of the experimental groups were covered with A-0 or A-5, and then fixed with a sticky 3M transparent dressing in each group. The morphology of wound was recorded and the wound area was measured at 0, 3, 7, 14, and 21 days.

Wound size (%) = (Wound area at a certain time)/(Initial wound area) × 100(10)

Mice were sacrificed at predetermined time points (7d), and the wound tissues were excised for in vivo antimicrobial testing, histological examination, and IHC analysis of VEGF, CD31, IL-6, and IL-10 [76].

## 4. Conclusions

We combined marine actinomycin X2 (Ac.X2) with silk fibroin (SF) through mild immobilization to prepare antimicrobial films (AMFs). These AMFs had enhanced antibacterial activity against *E. coli*, due to the higher density of Ac.X2 in the AMFs had increased inhibitory effects against *E. coli* and the ROS assay speculated that the AMFs caused apoptosis of the bacteria. Soluble Ac.X2 caused cytotoxicity, which was mainly due to the penetration of free Ac.X2 into the L929 cells causing cell death. Large amounts of Ac.X2 molecules were immobilized in the AMFs, which reduced the exudation of free Ac.X2 and reduced cytotoxicity. The determination of immobilization and exudation rate of Ac.X2 on the SF surface further explained that the combination of SF and Ac.X2 had advantage of reducing the free Ac.X2 in the system. Finally, the results of the full-thickness defect model repaired by the AMF treatment group showed that the wound shrinkage rate was higher, with denser collagen fibers and more complete epithelial tissues because Ac.X2 inhibited the bacterial growth on the wound tissue, improving the wound microenvironment. AMFs greatly promoted the expressions of CD31 and VEGF, crucial growth factors in the revascularization in wound healing as it is generally recognized that VEGF promotes revascularization in skin wound repair. In terms of biocompatibility, good biodegradability and hemocompatibility were demonstrated by the AMFs. Hence, the combination of Ac.X2 and biocompatible materials is a worthwhile consideration due to the enhanced antimicrobial and wound healing activities.

## Figures and Tables

**Table 1:** Immobilization density of different AMFs and corresponding amounts of leachates.

Sample	Immobilization Density (μg/cm^2^)	Immobilized Ac.X2 # (μg)	Free Ac.X2 Concentration # (μg/mL)	Amount of Leachates (ng/cm^2^)	Concentration of Leachates ## (ng/mL)
A-5	0.52	0.29	2.9	4.492	25.39
A-10	0.96	0.54	5.4	8.852	50.03
A-20	1.67	0.95	9.5	10.856	61.36

Note: Immobilization density is shown according to previous quantification. Exudative Ac.X2 solution was collected from AMF leachates at 24 h, then the content of Ac.X2 was analyzed by HPLC. # Indicates that the film was a disc with a diameter of 0.6 cm. ## The concentration of exudative Ac.X2 and 100 μL culture medium after homogenization was calculated for comparison of cell viability. Free Ac.X2 concentration #: The amount of the immobilized Ac.X2 on the 0.6 cm disc was converted into free Ac.X2 and then dissolved in 100 μL culture medium, which was used to estimate the cell survival rate, reflecting the advantages of immobilization.

## Supplementary Materials

The following supporting information can be downloaded at: https://www.mdpi.com/article/10.3390/ijms24076269/s1.
