# Supplementary material for: Actinomycin-X2-Immobilized Silk Fibroin Film with Enhanced Antimicrobial and Wound Healing Activities
Source: Int J Mol Sci. 2023 Mar 27;24(7):6269. doi: 10.3390/ijms24076269 (PMC10094675; doi:10.3390/ijms24076269)
Supplement: Supplementary file 1 [file ijms-24-06269-s001.zip › ijms-2264403-supplementary.pdf]

Supplementary Materials

Actinomycin-X2-Immobilized Silk Fibroin Film with Enhanced Antimicrobial and Wound Healing Activities

Wenjing Zhou [1], Zhenxia Xie [1], Ranran Si [2], Zijun Chen [1], Ansar Javeed [3], Jiaxing Li [1], Yang Wu [1], Bingnan Han [1,*]

[1] *Laboratory of Antiallergy Functional Molecules, Zhejiang Key Laboratory of Silkworm Bioreactor and Biomedicine, College of Life Sciences and Medicine, Zhejiang Sci-Tech University, Hangzhou 310018, PR China;*

[2] *School of Materials Science and Engineering, Zhejiang Sci-Tech University, Hangzhou, Zhejiang, 310018, China*

[3] *College of Textile Science and Engineering (International Institute of Silk), Zhejiang Sci-Tech University, Hangzhou, Zhejiang, 310018, PR China.*

* Corresponding author: hanbingnan@zstu.edu.cn

Content

Table S1. Initial concentrations of antimicrobial peptide Ac.X2 were used as IDs of AMFs……………………………………………………………………………..3

Figure S1. Physically absorbed Ac.X2 compared to covalently attached Ac.X2……………………………………………………………………………… .3

Figure S2. Representing transmittance of strongest absorption band…………………….5

Figure S3. Dissolve-loss rate and swelling rate of different AMFs at 37℃ for 7 days……………………………………………………………………………….5

Figure S4. Thermal analysis and tensile properties of AMFs…………………………….6

Figure S5. Results of initial screening stage of inhibitory concentration are shown…………………………………………………………………………….7

Figure S6. Micrographs of L929 fibroblast cell morphology on different AMFs……. 8

Figure S7. Viability curve of L929 fibroblasts cell at different concentrations of Ac.X2…………………………………………………………………………….8

Table S1. Initial concentrations of Ac.X2 were used as IDs of Ac.X2-immobilized antibacterial films (AMFs).

Sample IDs	A-0	A-1	A-5	A-10	A-20
The initial concentration of Ac.X2 (μg/ml)	0 (Control)	1	5	10	20

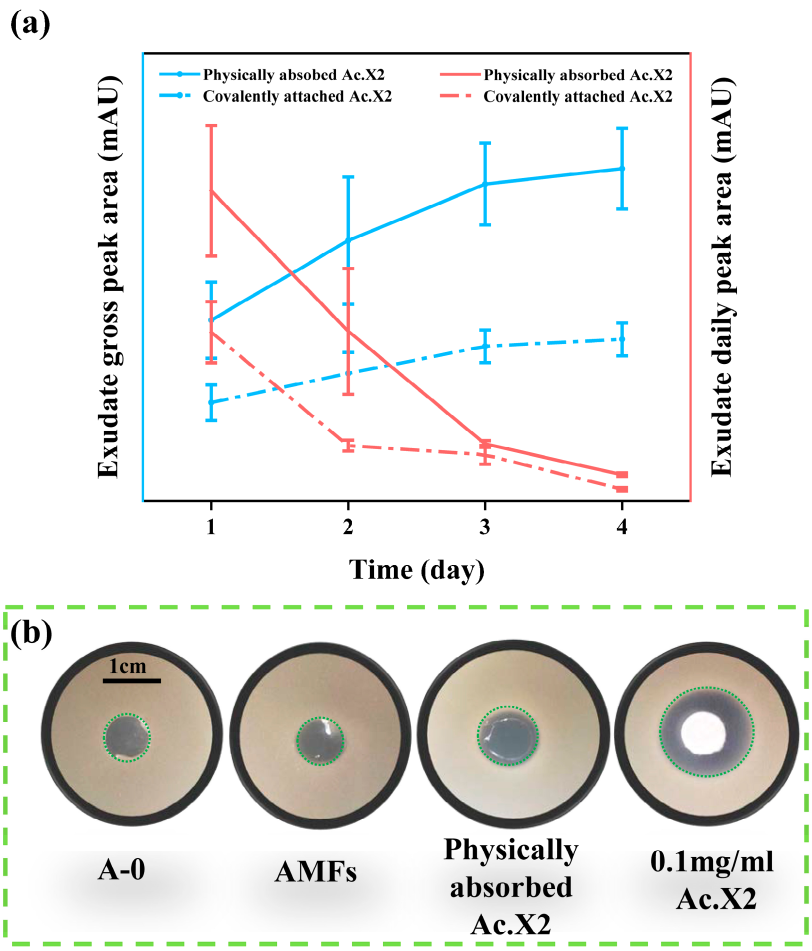

Figure S1. Physically absorbed Ac.X2 compared to covalently attached Ac.X2. (a) HPLC analysis of exudate transformed by the absorption intensity at 443 nm, whose peak area was shown within 4 days; (b) Inhibition zone of *S. aureus*, from left to right

was control, covalently attached AMPs (AMFs), physically absorbed AMPs and positive controls (Scale: 1 cm).

Comparison of different leachates. Verifying the difference between physically absorbed Ac.X2 (without EDC/NHS) and covalently attached Ac.X2 (AMFs), the leachates were obtained by shaking the SF film containing Ac.X2 at 180 rpm for 4 days. The peak area of the covalently attached Ac.X2 was lower than that of the physically adsorbed Ac.X2 (**Figure S1a**). This shows the advantage of covalent bonding and has a certain advantage in reducing the amount of exudative Ac.X2.

The existence of an antibacterial zone was a qualitative method to judge the antibacterial properties of target drugs. **Figure S1b** clearly shows that both the physically attached Ac.X2 and the positive control have the formation of a bacteriostatic zone, while the covalently attached Ac.X2 and the blank group have not visible bacteriostatic zone. The reason for this phenomenon was that the covalently attached Ac.X2 that cannot be freely diffused, which was not enough to produce effective bacteriostatic effect, while physically adsorbed Ac.X2 can reach this condition.

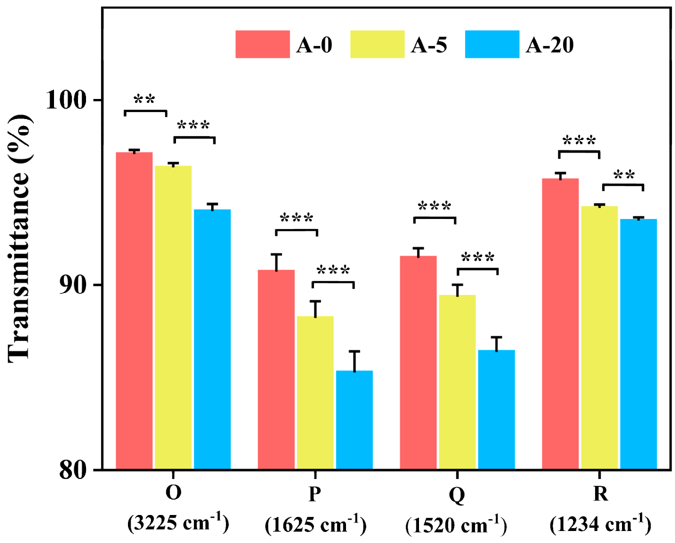

Figure S2. Representing transmittance of strongest absorption bands of O (3225 cm^{-1}), P (1625 cm^{-1}), Q (1520 cm^{-1}), R (1234 cm^{-1}). **Note:** ***$p < 0.001$, **$p < 0.01$.

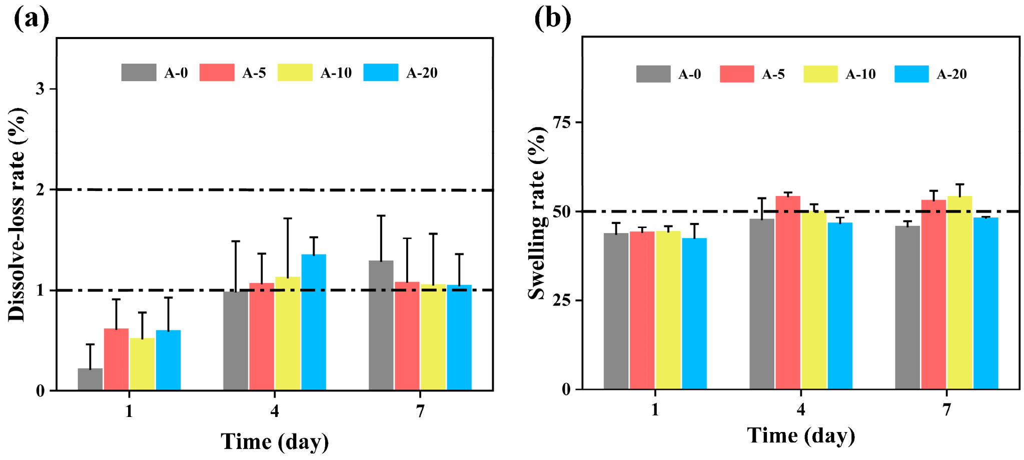

Figure S3. Dissolve-loss rate and swelling rate of different AMFs at 37 °C for 7 days.

Dried AMFs (W_1) were soaked in deionized water at 37 °C for 7 days and then AMFs were wipe dry and weighed (W_2); then it was dried in oven at 65 °C for 2 h and weighed again (W_3), each group has three parallel samples. Ratio of weight loss and swelling was calculated by following formula:

$$\text{Weight loss rate (\%)} = (W_1 - W_3) / W_1 \times 100$$

$$\text{Swelling rate (\%)} = (W_2 - W_1) / W_1 \times 100$$

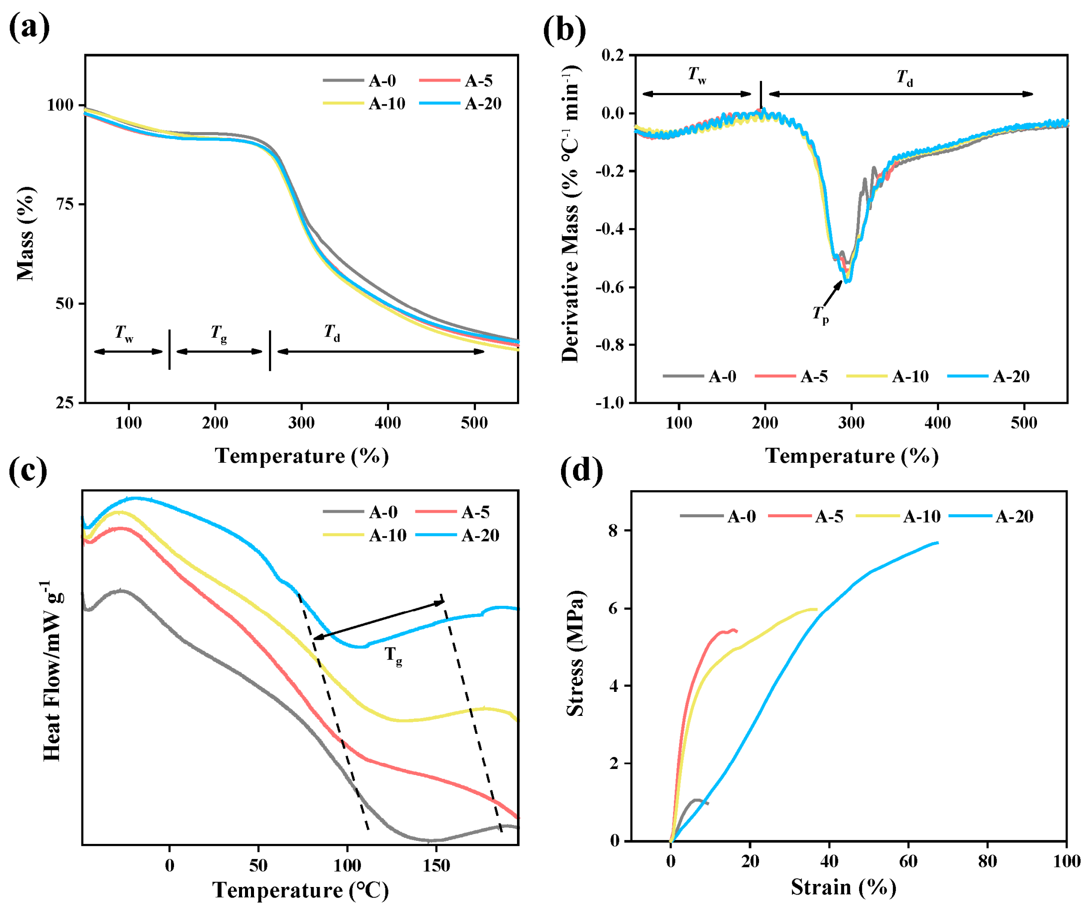

Figure S4. TGA (a), DTG (b) and DSC (c) curve of SF films with different Ac.X2, respectively. T_w, T_d, T_g represented dehydration, the glass transition and decomposition regions, T_p is the temperature at the maximum weight loss rate of the sample from DTG curve. (d) Stress–strain curves of SF films treated with different Ac.X2.

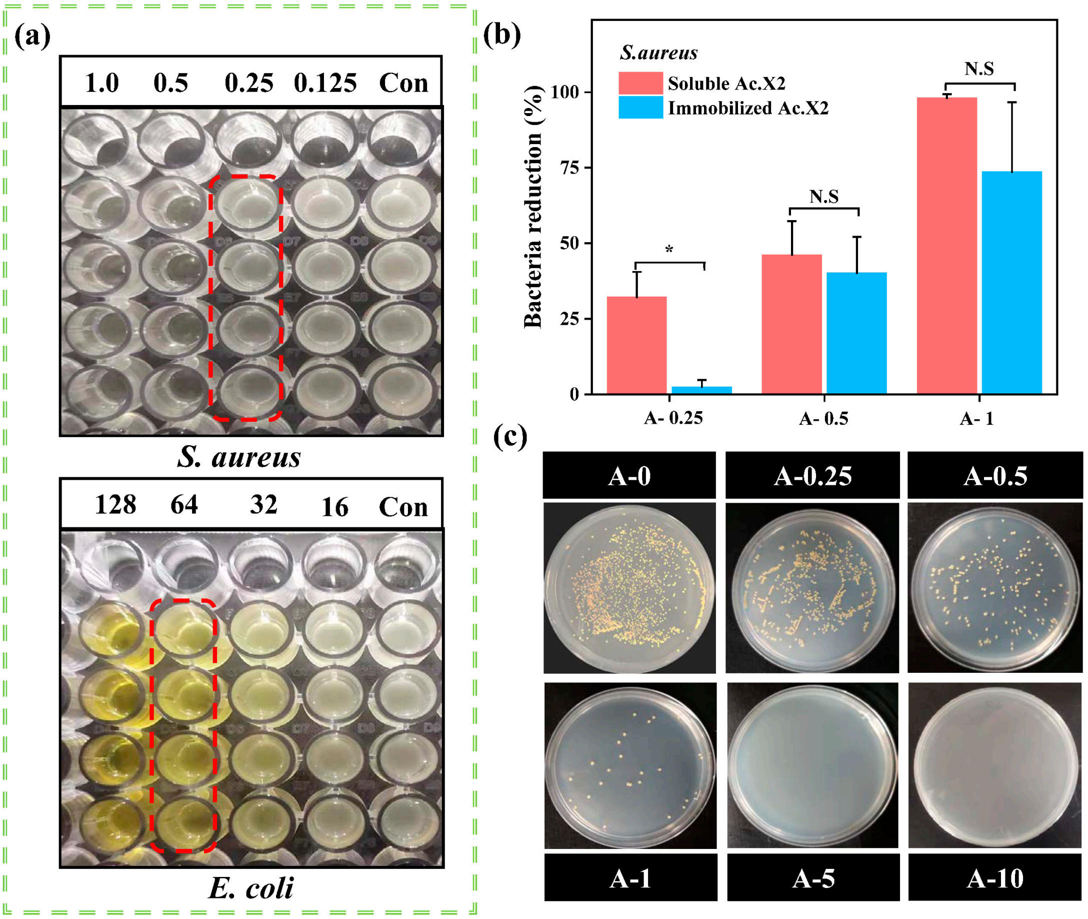

Figure S5. Results of initial screening stage of inhibitory concentration are shown. (a) Suspension density plot of bacteria detected by double dilution in 96-well plate at 600 nm; (b) *S. aureus* was inhibited, antibacterial activity of Ac.X2 with different concentration (0.25μg/ml, 0.5μg/ml, 1μg/ml) and same amount of immobilized Ac.X2 was compared; (* $p < 0.05$, N.S = Not Significant); (c) Colony map of *S. aureus* on agar plate treated with different AMFs for 24 h (from A-0 to A-10). **Note:** Colony map of A-20 was same as that of A-5 and A-10 was omitted.

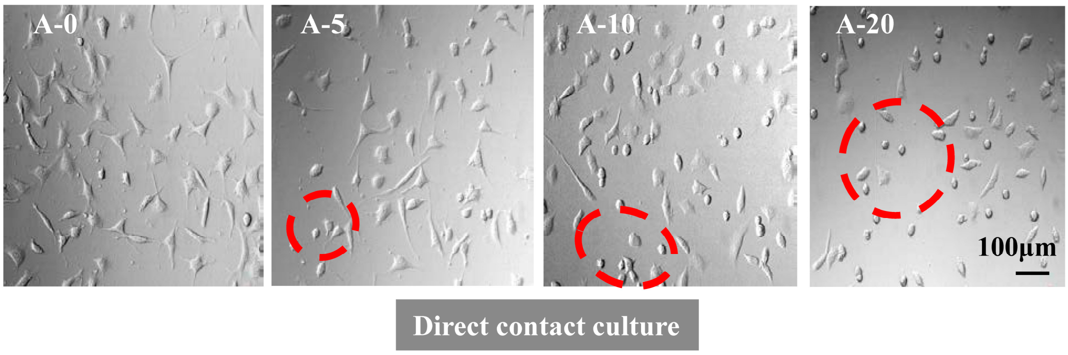

Figure S6. Micrographs of L929 fibroblast cell morphology on different AMFs by direct contact culture after 24 h, red marks represent abnormal cells (Scale: 100 μm).

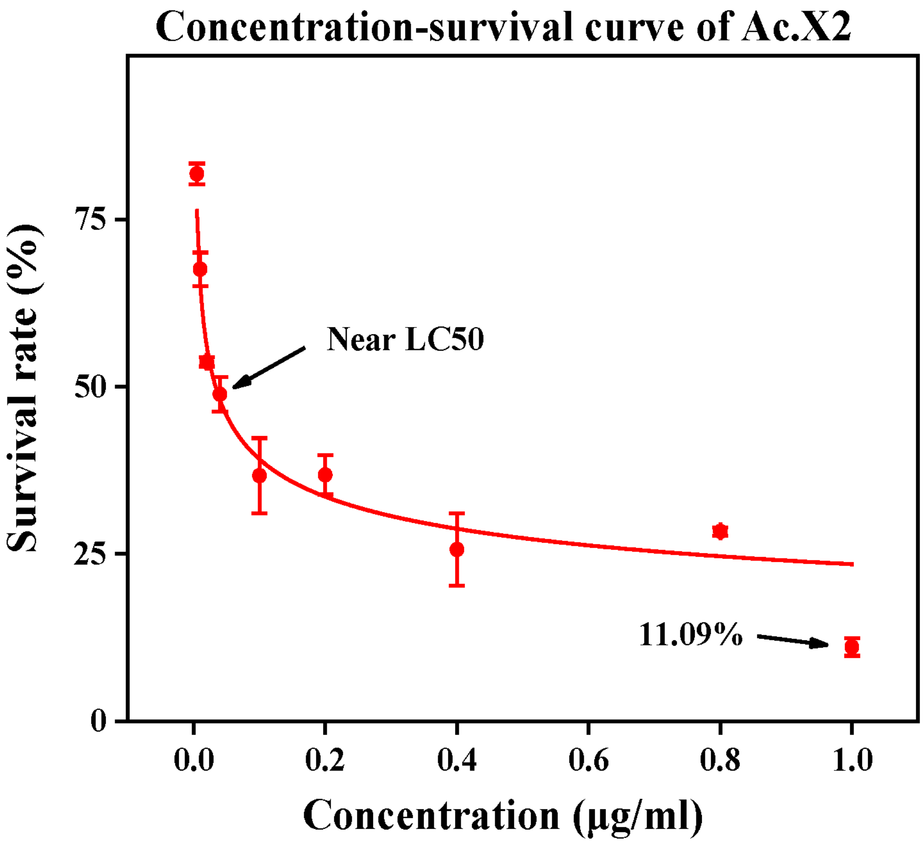

Figure S7. Viability curve of L929 fibroblast cells at different concentrations of free Ac.X2.
